# Additional Value of [18F]FDG PET/CT in Detection of Suspected Malignancy in Patients with Paraneoplastic Neurological Syndromes Having Negative Results of Conventional Radiological Imaging

**DOI:** 10.3390/jcm11061537

**Published:** 2022-03-11

**Authors:** Marta Opalińska, Anna Sowa-Staszczak, Kamil Wężyk, Jeremiasz Jagiełła, Agnieszka Słowik, Alicja Hubalewska-Dydejczyk

**Affiliations:** 1 Nuclear Medicine Unit, Department of Endocrinology, Oncological Endocrinology and Nuclear Medicine, University Hospital, 30-688 Kraków, Poland; mopalinska@su.krakow.pl; 2 Chair and Department of Endocrinology, Jagiellonian University Medical College, 31-008 Kraków, Poland; alahub@cm-uj.krakow.pl; 3 Department of Neurology, University Hospital, 30-688 Kraków, Poland; kwezyk@su.krakow.pl; 4 Chair and Department of Neurology, Jagiellonian University Medical College, 31-008 Kraków, Poland; jeremiasz.jagiella@uj.edu.pl (J.J.); agnieszka.slowik@uj.edu.pl (A.S.)

**Keywords:** paraneoplastic syndromes, neurooncology, [18F]FDG PET/CT, PNS

## Abstract

Background: Paraneoplastic neurological syndromes (PNS) affecting the CNS (central nervous system) are rare, presenting in less than 1% of all those with cancer. The pathogenesis of paraneoplastic neurological syndromes is not fully understood, but it is presumed to result from an immune attack on the underlying malignancy. The presence of different types of onconeural antibodies may occur in different tumors and can lead to different clinical manifestations, making the early detection of cancers challenging. Aim: An evaluation of [18F]FDG PET/CT in neoplastic tumor detection in patients with paraneoplastic neurological syndromes having negative or unremarkable results of conventional radiological imaging. Methods: Among all patients diagnosed with paraneoplastic neurological syndromes in the Neurology Department in 2016–2020, 15 patients with unremarkable conventional radiological findings who underwent [18F]FDG PET/CT were included in the study. Results: [18F]FDG PET/CT enabled localization of suspected malignancy in 53% (8 of 15) of PNS cases with previous unremarkable conventional radiological findings. Conclusion: [18F]FDG PET/CT may be considered as a useful tool for neoplastic tumor detection in patients with paraneoplastic neurological syndromes, accelerating the diagnostic process and enabling faster initiation of appropriate treatment.

## 1. Introduction

Paraneoplastic syndromes represent a wide range of symptoms associated with malignancy. They are usually systemic dysfunctions which are not a direct result of neoplastic tumor invasion or metastasis. They may manifest with symptoms from various systems and organs, causing endocrine, neurological, dermatological, rheumatological and hematological syndromes, usually, in areas and organs that are not directly affected by the neoplasm. The most common malignancies associated with paraneoplastic syndromes include breast, lung, pancreas, kidney as well as gynecological and hematological tumors. The incidence and prevalence of paraneoplastic disorders is estimated to be 0.89/100,000 person-years and 4.4 per 100,000, respectively [1]. In many cases, the etiology of the syndrome is unknown. Most often, they are autoimmune reactions to circulating tumor antigens or an effect of biologically active substances, such as hormones, growth factors or cytokines secreted by tumor cells.

Paraneoplastic neurologic syndromes (PNS) in the large majority respond well to the symptomatic therapy; however, not in all cases is such treatment effective. The population-based study estimated an incidence of PNS at about 1/100,000 person-years and a prevalence of 4/100,000, with the incidence ratio increasing over time [2]. The clinical presentation of PNS is excessively variable (Table 1); hence, the proper diagnosis requires extensive workup and clinical experience. In 2004, an international panel of experts recommended diagnostic criteria defining a neurological syndrome as a paraneoplastic syndrome based on the coexistence of onconeural antibodies and the presence of clinical symptoms. Further, they categorized those presentation as “classical” and “non-classical” syndromes. “Classical” syndromes (e.g., Lambert–Eaton myaesthenic syndrome, limbic encephalitis, encephalomyelitis, subacute cerebellar degeneration, sensory neuronopathy, dermatomyositis, or opsoclonus-myoclonus) are more likely to be associated with an underlying malignancy [3]. 

According to the current knowledge, PNS occur in two main mechanisms of immune response. The first is the direct immune response against neuronal receptors or other cell membrane antigens. The most common types of abovementioned antibodies and their clinical significance are summarized in Table 2. Those syndromes usually present clinically by rapidly developing neurological dysfunction, which might not be related to tumor size or growth and frequently respond well to anti-inflammatory treatment. In the second type, the immunoreactivity is triggered by intra-cellular neuronal proteins, which are more often associated with the development of malignancy. In that mechanism, the immune response targets originate from intracellular antigens/proteins revealed by neuronal death. In those conditions, neurological damage is usually more severe and very often irreversible. The most common types of antibodies against intra-cellular neuronal proteins, associated with underlying malignancy, and their clinical symptoms are summarized in Table 3. 

However, tumor removal itself is very seldom linked with good prognosis (e.g., teratoma removal in patients with NMDA (Anti-N-methyl-d-aspartate-mediated encephalitis) [4]), prompt identification and treatment are strongly recommended in order to eliminate proteins triggering the immune response (e.g., small cell carcinoma with anti Hu [5]). Within the pursuit of identifying the origin of malignancy, early imaging using radiological procedures (CT or MRI) often fails to establish the diagnosis. Among the widely available methods of advanced diagnostic procedures, a whole-body [18F]FDG PET/CT might be indispensable in the search for underlying pathology.

In the current state of knowledge, the value of [18F]FDG PET/CT as a pivotal imaging in doubtful cases is recommended but not fully elucidated. The rarity and complexity of neurological PNS leads to a paucity of information necessary to formulate strong diagnostic recommendations.

**Table 1:** Paraneoplastic syndromes of the nervous system classified by location. Adapted from Dalmau J, Rosenfeld MR. Paraneoplastic syndromes of the CNS. *Lancet Neurol*. 2008; 7(4): 327–340. [6].

	Brain, Cranial Nerves and Retina	Spinal Cord	Peripheral Nerves or Muscle	Neuromuscular Junction
Non-Classic PNS	Brainstem encephalitis	Stiff -person syndrome	Sensorimotor neuropathy	Myasthenia gravis
	Optic neuritis	Myelitis	Neuropathy and paraproteinaemia	
	Cancer-associated retinopathy	Necrotising myelopathy	Neuropathy with vasculitis	
	Melanoma-associated retinopathy	Motor-neuron syndromes	Polymyositis	
			Acute necrotising myopathy	
			Acquired neuromyotonia	
			Autonomic neuropathies	
Classic PNS	Cerebellar degeneration		Sensory neuronopathy	Lambert-Eaton myasthenic syndrome
	Limbic encephalitis		Intestinal pseudo-obstruction	
	Encephalomyelitis		Dermatomyositis	
	Opsoclonus-myoclonus

**Table 2:** Major paraneoplastic onconeuronal antibodies reactive with neuronal membrane antigens. Adapted from Galli, J.; Greenlee, J. Paraneoplastic Diseases of the Central Nervous System [7].

Antibody	Common Neurological Phenotypes	Common Associated Malignancies
Anti-AMPAR	Limbic encephalitis	Breast Lung Thymus
Anti-LGI1/Anti- CASPR2	Limbic encephalitis Faciobrachial dystonic seizures Morvan’s syndrome	Thymoma (especially in patients positive for both antibodies) Other neoplasms (rare)
Anti-GABAbR	Limbic encephalitis, status epilepticus	Small-cell lung cancer
Anti-mGluR1	Cerebellar degeneration	Hodgkin’s disease
Anti-mGlur2	Cerebellar degeneration	Small-cell cancer; alveolar rhabdomyosarcoma
Anti-mGluR5	Limbic encephalitis	Hodgkin’s disease
Anti-VGKC	Cerebellar degeneration (Lambert–Eaton myasthenic syndrome)	Small-cell lung cancer

**Table 3:** Major paraneoplastic onconeuronal antibodies reactive with intracellular neuronal antigens. Adapted from Galli, J.; Greenlee, J. Paraneoplastic Diseases of the Central Nervous System [7].

Antibody	Common Neurological Phenotypes	Common Associated Malignancies
Anti-CRMP5	Optic neuritis Cerebellar degeneration Encephalomyelitis	Small-cell lung cancer Breast carcinoma
Anti-GAD65	Stiff person syndrome Limbic encephalitis Cerebellar ataxia	Thymoma Renal cell carcinoma
Anti-Hu (ANNA-1)	Limbic encephalitis, encephalomyelitis, dorsal sensory neuropathy	Small-cell lung cancer Neuroendocrine tumors Retinoblastoma (infants)
Anti-Ma1	Limbic or brain-stem encephalitis	Non-small-cell lung cancer; other
Anti-Ma2	Limbic or brain-stem encephalitis	Testicular or other germ cell tumors Non-small-cell lung cancer
Anti-Ri (ANNA-2)	Cerebellar degeneration, opsoclonus myoclonus, brain-stem encephalitis	Breast Small-cell lung cancer
Anti-Tr	Cerebellar degeneration	Hodgkin’s disease
Anti-Yo (PCA-1)	Cerebellar degeneration	Ovary, uterus, adnexa Breast

The aim of the study was to assess the usefulness of [18F]FDG PET/CT in patients with high risk of PNS and negative or unremarkable results of conventional radiological imaging.

## 2. Methods

Among the patients diagnosed with neurological syndromes between 2016–2020 in the Neurology Department of University Hospital in Krakow, we selected cases with a clinical picture strongly suggesting an underlying neoplastic background (rapid course of PNS and resistance to conventional first-line treatment). 

In 15 of them, with positive or unremarkable results of onconeuronal antibodies, due to negative or unambiguous results of conventional screening (including radiological and endoscopy workup), the [18F]FGD PET/CT was applied.

Among these patients, seven were diagnosed with classical neurological PNS (cerebellar degeneration—3 cases, sensory polyneuropathy—1 case, autoimmune encephalitis—3 cases), and 7 with non-classic PNS (myasthenia gravis (MG)—1, myelitis—1, motor neuron disease—1, sensorimotor polyneuropathy—4). Additionally, a patient with Primary Angiitis of Central Nervous System (PACNS) (1) was included in the analysis, since several cases of sporadic presentation of that disease as a paraneoplastic syndrome are available in the literature. 

All patients’ demographic data, clinical presentation, cerebrospinal fluid (CSF) evaluation, previous imaging results and onconeuronal antibodies screening were recorded from the available patient database. The distribution of PNS types diagnosed among the analyzed group is presented in Table 4.

### 2.1. Image Acquisitions and Analysis

[18F]FDG PET/CT examinations were conducted in accordance with the standard protocol on GE DISCOVERY 690 VCT scanner (Krakow, Poland). All patients fasted at least 6 h before the procedure. Bedside fasting blood glucose of more than 11.0 mmol/L was not accepted. Imaging was performed 60 min after intravenous administration of the radiotracer. An initial low-dose CT without contrast enhancement (Smart mA with range: 50–180 mA) was performed, in order to correct for photon attenuation and to co-localize FDG uptake and anatomical structures. The FDG PET/CT was performed from the mid-thigh to the top of the head. Patients received 4 MBq [18F]FDG per 1 kg body mass (2 min per bed position in three-dimensional mode). The PET data were reconstructed with the use of the GE (matrix size 128 × 128, Vue Point FX reconstruction method: 16 subsets, 3 iterations reconstruction algorithm). Corrections were applied for attenuation, dead time, scatter and random coincidences. All image analyses were performed on fused PET/CT data sets. Slice thickness of the PET short-axis images was 3.3 mm. The estimated dose of radiation per patient was about 9.5 mSv.

All images were reviewed using first attenuation-corrected images. In all cases, a senior nuclear medicine consultant made an initial assessment of [18F]FDG-PET scans, which was then re-assessed by the consultant radiologist.

The PET/CT was binary classified, so a positive result was defined as the presence of a metabolically active tumor of any region of body, suspicion of metabolically active dissemination of neoplastic disease or increased focal uptake, being highly suspected to correspond with malignancy.

The negative results were considered as scans with no FDG uptake, and with any focal increased uptake with intensity higher than the surrounding tissues, but in localization not highly suspicious as a malignancy.

### 2.2. Statistical Analysis

Demographic and clinical characteristics were analyzed by producing tables of frequency for categorical variables and by calculation of the median and range for continuous variables. IBM SPSS Statistics for Windows, version 27 (IBM Corp., Armonk, NY, USA) was employed for the statistical analysis.

## 3. Results

A group of 15 patients (9 females and 6 males) were eligible for the study. The median age was 70 years (range: 32–88 years). All patients underwent anatomical neuroimaging of the brain—in 5 cases, head CT and in the remaining cases, MRI. A total of 14 patients also underwent lumbar puncture; in one patient, due to lack of consent, only blood tests were performed. The whole group was evaluated for the presence of onconeuronal antibodies [Table 5]. Positive results of [18F]FDG PET/CT were found in 8 out 15 cases, which were associated with 4 types of neurological PNS. 

The following percentages of positive results were found: in patients with myelitis 100% (1 case), cerebral degeneration 75% (3 of 4 cases) and among patients with polyneuropathies 50% (2 of 4 cases). 

**Figure 1:**
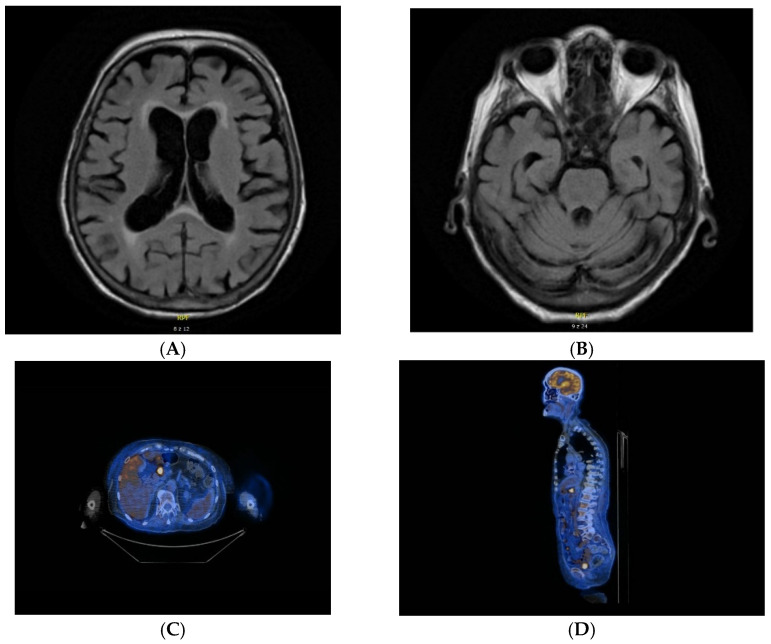
MRI and PET/CT results of a patient with cerebellar degeneration. (**A**,**B**) nonspecific vascular demyelination detected on MRI. (**C**,**D**) metabolically active area in the pylorus and in the locoregional lymph nodes detected on [18F]FDG PET/CT.

Among patients with myasthenia gravis, primary angiitis of the central nervous system and motor neuron disease there were no positive results of PET/CT (total number of observations: 4). The increased FDG uptake was found in 4 cases in lungs, in 1 case in colon, in 2 cases in lymph nodes of neck and chest and in 1 case in stomach (Figure 1).

The mean value of SUV max in pathological findings were 6.59 (range 2.2–10.5). 

The [18F]FDG PET/CT findings along with patients’ demographic information, clinical presentation, results of brain/spinal cord imaging, presence and type of onconeural antibodies, and CSF analysis results are summarized in Table 5.

## 4. Discussion

PNS are rare (less than 1%) manifestation of malignancy. However, its significance is greater, because of diagnostic difficulties and demanding therapeutic process. Due to the increased availability of onconeural antibody tests, there is a progress in both early diagnosis and the implementation of effective targeted therapy.

According to published guidelines for PNS diagnosis [8] in all cases tumor removing is indicated, increasing chance for proper control of PNS symptoms. In patient (No. 4), the neurological symptoms including involuntary movements and psychotic symptoms, were refractory to therapy with steroids, intravenous immunoglobulins and monoclonal anti-CD20 antibody. Only identification and resection of the papillary thyroid cancer led to remission, however PET/CT examination revealed dissemination.

Other risk factors of neurological autoimmune disorders such as coexisting autoimmune disease or a family history of autoimmunity may mislead the initial diagnosis, but in all cases, exclusion of malignancy is mandatory. The evaluation of serum cancer markers such as CA-125, CA-15.3, prostate-specific antigen, and carcinoembryonic antigen with subsequent CSF assessment are usually the first steps in diagnostic workup. In our cohort, only one patient (No. 10) had an elevated carcinoma antigen 15-3 (Ca 15-3), suggesting an underlying paraneoplastic syndrome; however, the [18F]FDG PET/CT result was negative. 

In many patients, CSF analysis demonstrates abnormalities such as elevated protein, pleocytosis, the presence of oligoclonal bands or mixed abnormalities, but normal CSF analysis results do not exclude the possibility of PNS [9]. Patients No. 2, 4 and 14 had normal CSF findings, although other elements of the clinical picture indicated the possibility of PNS. Finally, only in the patient with cerebellar syndrome (No. 2) did the PET/CT scan show metabolically active lymph nodes in the mediastinum and chest, suggesting myeloproliferative syndrome. 

Onconeuronal antibodies may act as markers for the specific tumors, e.g., anti-Yo for breast or reproductive system tumors, anti-Hu for small-cell lung cancer, anti-Ri for breast or lung or anti-Ma2 for testicle malignancy. In these cases, targeted tumor seeking by CT, mammography, pelvic or testicular ultrasound is essential. However, in cases with high risk of malignancy established by the presence of antibodies directed against intra-cellular neuronal proteins and adequate clinical presentation, PET/CT with [18F]FDG may bring additional benefits due to the high sensitivity of PET/CT in the detection of small, aggressive tumors. Furthermore, many types of PNS and onconeuronal antibodies can appear in several types of tumors, such as Anti-Yo antibodies which were found in patients with cerebellar syndrome (No. 1, 2), primary CNS vasculitis (No. 14) and motor neuron disease (No. 15). In this context, in cases with positive, unspecific for particular malignancy, antibodies, PET/CT may have additional value, enabling whole-body screening during a single examination.

The meta-analysis evaluating the suitability of [18F]FDG PET/CT in PNS demonstrated a pooled sensitivity of 0.81 and specificity of 0.88 in patients with suspicion of paraneoplastic syndrome [10]. However, that high pooled sensitivity results from data analysis of all patients with PNS having both positive and inconclusive/negative results of prior conventional imaging. In our study, the efficacy of [18F]FDG PET/CT was 53%, but only patients with negative previous radiological and endoscopic exams were evaluated in whom PET/CT had a real clinical benefit.

In the clinical context, it is worth considering if and when PET/CT should be repeated in case of a negative initial study. The guidelines recommend regular oncological surveillance every 6 months for up to 4 years [11] for initially negative imaging results; however, prolonged time of observation may be connected with unfavorable outcomes in some patients. 

The worsening of a known autoimmune syndrome or the occurrence of a new one may suggest dissemination or recurrence of the malignancy. In two patients (No. 3 and 8), the PET/CT exam was repeated (after 12 and 3 months, respectively) due to progressing clinical symptoms. In patient 8 the second exam revealed lung malignancy. 

Several studies have shown that whole-body [18F]FDG-PET and [18F]FDG PET/CT are very useful in the screening of patients with suspected paraneoplastic syndrome and positive paraneoplastic antibodies [12]. However, a recent study conducted by another group suggested that the presence of paraneoplastic antibody should not be an indispensable factor for performing [18F]FDG PET/CT [13,14]. In our study, the effectiveness of [18F]FDG PET/CT in the detection of pathology in patients with the presence and absence of onconeuronal antibodies was confirmed. In three PNS patients with negative onconeural antibodies and clinical presentation highly suggesting malignancy, PET/CT scans were positive. Furthermore, in total, there were 53% of positive [18F]FDG PET/CT results (50% and 57% of patients with non-classical and classical PNS, respectively). 

The results of our study indicate the potential benefits of [18F]FDG PET/CT in a routine neurological practice in selected patients with PNS, regardless of their onconeural antibody status and type of PNS.

## 5. Highlights

[18F]FDG PET/CT may be effective in malignancy detection in a large group of PNS patients with negative results of prior diagnostic procedures.

In a routine neurological practice, selected patients with PNS may benefit from [18F]FDG PET/CT regardless of their onconeural antibody status and type of PNS.

Repeating [18F]FDG PET/CT may have additional value, but the optimal timing for the second examination remains uncertain.

## Figures and Tables

**Table 4:** The distribution of PNS types diagnosed among the analyzed group and the results of [18F]FDG examination.

Classic PNS	No of Patients with PNS Included to Analysis	No of Patients with Positive [18F]F-FDG PET/CT Findings	Percentage of PET/CT Positive Examination
Cerebellar degeneration	3	3	100
Sensory polyneuropathy	1	0	0
Autoimmune encephalitis	3	1	33
Non-classic PNS			
Myasthenia gravis	1	1	100
Myelitis	1	1	100
Sensorimotor polyneuropathy	4	2	50
Motor neuron disease	1	0	0
Others			
Primary Angiitis of Central Nervous System (PACS)	1	0	0
Sum	15	8	53

**Table 5:** Clinical. biochemical. and imaging data of patients included in the study.

Patient No	Sex, Age	Neuroimaging (CT, MR)	CSF Results	Antibodies	Metabolic Abnormalities of CNS	PET/CT Abnormalities
Cerebellar Degeneration						
1.	F. 88	Brain MRI: nonspecific vascular demyelination; no clinical relevance	Cytosis 0.005 × 10^3^/uL (0–0.005 × 10^3^/uL) Protein 48.5 mg/dL (20.00–40.00) oligoclonal bands: positive	anti-Yo	none	metabolically active area in the pylorus and metabolically active lymph node next to the pylorus SUV max 7.8
2.	F. 83	Brain MRI: leukoaraiosis around lateral ventricles. nonspecific vascular demyelination. moderate cortical artrophy—mainly posterior. bilateral hyperintense changes in the thalami and caudate nuclei on diffusion weighted imaging (DWI).	Cytosis 0.004 × 10^3^/uL (0–0.005 × 10^3^/uL) Protein 34.8 mg/dL (20.00–40.00) oligoclonal bands: negative	anti-NMDA. anti-Yo	Generalized cortico-subcortical atrophy of the brain.	metabolically active lymph nodes in the mediastinum and chest—suspection of lymphoma SUV max 10.5
3.	M. 66	Brain MRI: not performed (due to contraindications) Brain CT: normal	Cytosis 0.006 × 10^3^/uL (0–0.005 × 10^3^/uL) Protein 40.6 mg/dL (20.00–40.00) oligoclonal bands: positive	not detected	not detected	metabolically active tumor in the transverse colon SUV max 5.0
Autoimmune Encephalitis						
4.	F. 32	Brain MRI: normal	Cytosis 0.018 × 10^3^/uL (0–0.005 × 10^3^/uL) Protein 26.3 mg/dL (20.00–40.00) oligoclonal bands: negative	anti-NMDA	none	increased metabolism of FDG in the topography of numerous lesions in both lungs SUV max up to 7.5
5.	M. 30	Brain MRI: demyelinating lesions located bilaterally in the white matter of the frontal and parietal lobe, in the periventricular and subcortical areas.	Cytosis 0.002 × 10^3^/uL (0–0.005 × 10^3^/uL) Protein 47.2 mg/dL (20.00–40.00) oligoclonal bands: negative	anti-NMDA	none	not significant
6.	F. 74	Brain MRI: epidermal cysts located anteriorly from the medulla oblongata, on the left side; numerous demyelinating lesions located in the white matter of the centrum semiovale, periventricular and paraventricular areas; numerous, small signalless zones on Susceptibility-Weighted Imaging (SWI) which correspond to the presence of haemosiderin deposits—after microchemorrhages	Cytosis 0.002 × 10^3^/uL (0–0.005 × 10^3^/uL) Protein 37.3 mg/dL (20.00–40.00) oligoclonal bands: positive	anti-NMDA	cortical-subcortical atrophy of the brain	not significant
Myasthenia Gravis						
7.	F. 79	Brain MRI: nonspecific vascular demyelination; no clinical relevance	not performed	anti-AChR	areas of porencephaly with decreased FDG metabolism	not significant
Myelitis						
8.	F. 70	Brain MRI: leukoaraiosis around lateral ventricles; few. small changes with increased signal in T2 in the radial corona radiata in the frontal lobes; moderate cortical atrophy of the brain and cerebellum. Cervical Spine MRI: The zone of increased signal in the T2 sequences dependent on the central part of the spinal cord. extending from the C2 / C3 to C6 / C7 level	Cytosis 0.008 × 10^3^/uL (0–0.005 × 10^3^/uL) Protein 74 mg/dL (20.00–40.00) oligoclonal bands: negative	anti-AQP4	none	moderately metabolic active tumour in segment 4/5 of the right lung. SUV max 3.5
Polyneuropathy						
9.	M. 59	Brain MRI: not performed. Brain CT: normal	not performed	not detected	none	metabolically active tumor in the apex of the left lung with the involvement of homonymous mediastinal lymph nodes—SUV max 9.4
10.	F. 56	MRI: not performed. Brain CT: normal	not performed	not detected	none	not significant
11.	F. 73	Brain MRI: not performed. Brain CT: normal	Cytosis 0.003 × 10^3^/uL (0–0.005 × 10^3^/uL) Protein 144.1 mg/dL (20.00–40.00) oligoclonal bands: negative	anti-PNMA2 (Ma2/Ta) anti-CV2.1	None	metabolically active cervical lymph node and a metabolically active soft tissue mass in the lower part of the neck and in the upper mediastinum SUV max 5.7
12.	M. 69	Brain MRI: nonspecific vascular demyelination; no clinical relevance.	not performed	anti-PNMA2 (Ma2/Ta)	none	not significant
13.	M. 65	Brain MRI: nonspecific vascular demyelination; no clinical relevance.	Cytosis 0.002 × 10^3^/uL (0–0.005 × 10^3^/uL) Protein 187 mg/dL (20.00–40.00) oligoclonal bands: negative	not detected	none	lesion in the 1 + 2 segment of left lung SUV max 2.2
Primary Angiitis of Central Nervous System						
14.	M. 38	Brain MRI: disseminated demyelinating lesions located in the cortico-subcortical area of the right insula. bilaterally in the corona radiata. and single irregular lesions with cortico-subcortical distribution.	Cytosis 0.004 × 10^3^/uL (0–0.005 × 10^3^/uL) Protein 38.2 mg/dL (20.00–40.00) oligoclonal bands: negative	anti-Yo	none	nonspecific segmental metabolic stimulation in the loops of the small intestine SUV max 7.7
Motor Neuron Disease						
15.	F. 67	Cervival Spine MRI: multi-level discopathy without pressure on the surrounding nerve roots.	Cytosis 0.001 × 10^3^/uL (0–0.005 × 10^3^/uL) Protein 67.96 mg/dL (20.00–40.00) oligoclonal bands: negative	anti-Yo	none	not significant

## Data Availability

All data generated or analyzed during this study are included in this article. Further enquiries can be directed to the corresponding author.
